# Supplementary material for: The Effect of a Combination of Eucommia ulmoides and Achyranthes japonica on Alleviation of Testosterone Deficiency in Aged Rat Models
Source: Nutrients. 2022 Aug 15;14(16):3341. doi: 10.3390/nu14163341 (PMC9414994; doi:10.3390/nu14163341)
Supplement: Supplementary file 1 [file nutrients-14-03341-s001.zip › nutrients-1842422-supplementary.pdf]

Table S1. Sequences of Primers Used for Real-Time RT-PCR Amplification.

Target gene	Primer sequences (5′→3′)	Product length (bp)	Accession No.
Cyp11a1	Forward: 5′-CTTTGGTGCAGGTGGCTAG-3′<br>Reverse: 5′-CGGAAGTGCGTGGTGTTT-3′	115	XM_039080972.1
Hsd3b1	Forward: 5′-TGTGCCAGCCTTCATCTAC-3′<br>Reverse: 5′-CTTCTCGGCCATCCTTTT-3′	145	NM_001007719.3
Hsd17b3	Forward: 5′-GACCGCCGATGAGTTTGT-3′<br>Reverse: 5′-TTTGGGTGGTGCTGCTGT-3′	140	NM_054007.1
Srd5a2	Forward: 5′- GGCAGCTACCAACTGTGACC-3′<br>Reverse: 5′- CTCCCGACGACACACTCTCT-3′	159	NM_022711.5
Cyp19a1	Forward: 5′-GCCTGTCGTGGACTTGGT-3′<br>Reverse: 5′-GGTAAATTCATTGGGCTTGG-3′	142	NM_017085.3
β-Actin	Forward: 5′-TCGTGCGTGACATTAAAGAG-3′<br>Reverse: 5′-ATTGCCGATAGTGATGACCT-3′	134	NM_031144.3

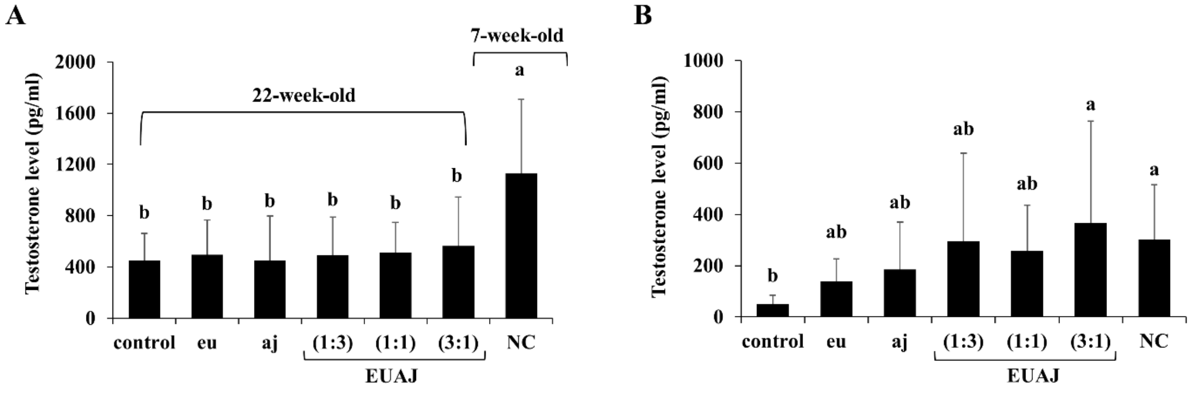

Figure S1. The Effects of EUAJ on testosterone levels in serum of SD rats. Serum testosterone levels of SD rats administered by eu, aj and its combinations in various proportions were shown in (A) and (B). Values are presented as means ± SD. Different superscript letters show a significantly difference at $p < 0.05$ as determined by Duncan's multiple range test. Control; saline, aj:aj (40 mg/kg), eu:eu (40 mg/kg), EUAJ (1:3); eu:aj=1:3 (40 mg/kg), EUAJ (1:1); eu:aj=1:1 (40 mg/kg), EUAJ (3:1); eu:aj=3:1 (40 mg/kg).

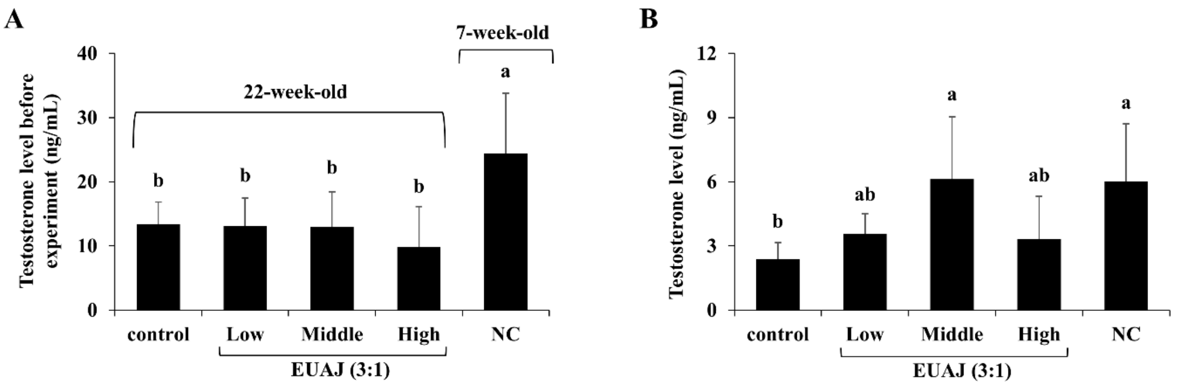

Figure S2. The Effects of various concentration of EUAJ on testosterone levels in serum of SD rats. Serum testosterone levels of SD rats were shown in (A) before administered by EUAJ (3:1). The level of (B) total testosterone of SD rats oral gavage of various concentration of EUAJ (3:1) for 6 weeks. Values are presented as means ± SD. Different superscript letters show a significantly difference at $p < 0.05$ as determined by Duncan's multiple range test. control; saline, Low; EUAJ (3:1) 20 mg/kg, Middle; EUAJ (3:1) 40 mg/kg, High; EUAJ (3:1) 80 mg/kg, NC; saline.
